# The Impact of Exercise Interventions on the Network Structure of Psychotic Symptoms: Analysis From Two Clinical Trials

**DOI:** 10.1111/eip.70124

**Published:** 2026-01-07

**Authors:** Kim Laurendeau, Paquito Bernard, Florence Piché, Amal Abdel‐Baki, Ahmed Jérôme Romain

**Affiliations:** ^1^ Centre de recherche de l'Institut Universitaire en Santé Mentale de Montréal (CRIUSMM) Montréal Québec Canada; ^2^ Univ Rennes, Inserm, EHESP, Irset (Institut de Recherche en Santé, Environnement et Travail)—UMR_S Rennes France; ^3^ École de kinésiologie et des sciences de l'activité physique (EKSAP), Faculté de médecine Université de Montréal Montréal Québec Canada; ^4^ Centre de recherche du Centre hospitalier de l'Université de Montréal (CHUM) Montréal Québec Canada; ^5^ Département de psychiatrie et addictologie, Faculté de médecine Université de Montréal Montréal Québec Canada

**Keywords:** mental health, obesity, physical activity, psychotic disorders

## Abstract

**Background and Hypothesis:**

In people with psychotic disorders, exercise is known to improve psychotic symptoms; however, the mechanisms underlying these effects are unclear. In the network approach, mental disorders are conceptualised as complex systems of interacting symptoms. In this context, exercise interventions could modify the dynamic of psychotic symptoms within the network. Using data from two independent clinical trials using exercise, the aim was to investigate the impact of exercise interventions on network connectivity, then compare the network structure pre and post intervention.

**Study Design:**

Combined data from two clinical trials on exercise with a total of 106 participants with a diagnosis of psychotic disorder were included. The Positive and Negative Syndrome Scale (PANSS) was used to assess symptom severity using semi‐structured interviews. Networks analyses were performed to compare before and after exercise.

**Study Results:**

At baseline, the PANSS network was densely connected with several strong positive connections. Symptoms being most central were negative symptoms. After exercise, the network was less dense and less connected, and the connections were different. When the networks before and after exercise were compared, they were significantly different in terms of structure, but not global strength.

**Conclusion:**

This study is the first to show that exercise seems to favour a disconnection between psychotic symptoms and could modify the network structure, providing a first mechanism of action which would require more investigation.

## 1 Introduction

Psychotic disorders are often chronic illnesses marked by positive, negative, disorganised and cognitive symptoms, leading to severe functional impairment that significantly limits major life activities (National Institute of Mental Health 2022).

To better understand symptoms in people with psychotic disorders, the network theory of mental disorders proposes an alternative framework where symptoms are a network of reciprocal influences (Borsboom 2017). This model of psychopathology follows four principles: (1) complexity (i.e., interactions between different components in a psychopathology network enhance the characterisation of mental disorders), (2) direct causal connections between symptoms, (3) mental disorders have a network structure and (4) hysteresis (i.e., symptom(s) or intervention (dis)activate the connection between symptoms, and the network can become self‐sustaining due to feedback associations). Besides, the network theory suggests that a stable mental state (characterised by being asymptomatic, or having fewer or less severe symptoms) is represented by a weakly connected network, whereas more symptomatic states are represented by a strongly connected network (Borsboom 2017; Borsboom and Cramer 2013).

In people with psychotic disorders, one study (Peralta et al. 2020) examining the cross‐sectional network structure of psychotic symptoms found a highly connected network structure, with disorganisation and negative symptoms emerging as the most central. A second cross‐sectional study among adults with schizophrenia showed that remitted participants (those with no/fewer symptoms) displayed sparser networks compared to non‐remitted participants, a pattern also observed in treatment‐responsive versus treatment‐resistant participants (Esfahlani et al. 2018). These studies support the network theory assumption that more severe symptoms correspond to dense, highly connected networks in people with psychotic disorders, whereas interventions (e.g., antipsychotics) can lead to sparser networks of symptoms. However, contrasting results have been found, particularly in intervention studies (Esfahlani et al. 2017; Strauss et al. 2019), where participants who responded to their medication also had an increased connectivity among networks of symptoms. Similarly, another study (Strauss et al. 2019) found that more densely connected symptom networks could also be associated with less severe symptoms among people with psychotic disorder.

Among interventions used to manage symptoms in people with psychotic disorders, exercise has been found to be clinically effective for people with psychotic disorders. Most exercise modalities (interval training, endurance training) have been shown to improve positive, negative, and general psychopathology symptoms as well as global and social functioning (Dauwan et al. 2016; Firth et al. 2015; Romain et al. 2019; Stanton and Happell 2014; Vancampfort et al. 2012; Wu et al. 2015). However, despite its efficacy, the mechanisms underlying the effects of exercise interventions on symptoms improvement remain unclear. One potential mechanism is that exercise can reduce the connectivity between the symptoms, as suggested by the network theory, thereby lowering symptoms severity. However, to date, no studies have specifically investigated this hypothesis.

Therefore, the aim of the present research was to investigate the impact of exercise interventions on psychotic symptoms from a network perspective. It was hypothesised that exercise would weaken symptoms connections, resulting in a sparser and less connected network of psychotic symptoms.

## 2 Methods

The present study used data from two clinical trials, which was possible because data regarding exercise and psychiatric symptoms were collected and assessed by the same evaluators (A.J.R. and research assistants) and using the same standardised procedures.

### 2.1 Study 1

The first study was a randomised controlled trial including 66 participants with psychotic disorders (for details, see Romain et al. 2019), and whose primary outcome was waist circumference. Briefly, the inclusion criteria were: (1) being adult (18–55 years old), (2) diagnosis of psychotic disorder according to the DSM‐IV‐TR by a psychiatrist, (3) taking antipsychotic medication, (4) overweight or obesity, (5) meeting the criterion for metabolic syndrome, (6) being inactive, (7) symptoms of psychotic disorder being stable for over a month and (8) no expected changes in medication. Patients with absolute contraindications to exercise and substance abuse were excluded.

#### 2.1.1 Intervention

Participants were randomised in two groups: the intervention group had a 6‐month supervised exercise intervention (supervised interval training, 30 min, twice per week) and the control group was in usual care. After the 6‐month period, participants from the control group also benefited from the exercise intervention from the sixth to the twelfth month (Romain et al. 2019).

#### 2.1.2 Results on PANSS Scale

In Study 1, in the intervention group, positive symptoms significantly decreased by 1.15 points, negative symptoms by 2.7 points and general psychopathology by 5.4 points.

### 2.2 Study 2

The second study was an open clinical trial including 40 participants with first episode or early psychosis recruited in an early psychosis service. Complete information is available in Dubois et al. (2020). Briefly, the inclusion criteria were: (1) being treated in an early psychosis service (18 and 30 years old), (2) diagnosis of psychotic disorder according to the DSM‐IV‐TR established by a psychiatrist, (3) symptoms of psychotic disorders being stable for over a month and (4) being physically inactive. Patients with absolute contraindication to exercise, pregnant women, or women planning to become pregnant were excluded.

#### 2.2.1 Intervention

Open trial where participants had a 6‐month exercise intervention (45 min, twice per week, supervised by exercise professionals). The intervention was based on participants' preferences (including a variety of exercise) (Romain et al. 2020; Dubois et al. 2020), included a motivational intervention to improve adherence, and the main outcome was feasibility and acceptability.

#### 2.2.2 Results on PANSS Scale

In Study 2, across the intervention, positive symptoms significantly decreased by 1.12 points, negative symptoms by 3.08 points and general psychopathology by 2.92 points.

### 2.3 Data Merging

The two studies were merged because they shared substantial similarities in their sample and design. In the two studies, participants had a diagnosis of psychotic disorder, were taking antipsychotic medication, had stable symptoms, and were physically inactive. Regarding the interventions, exercise sessions were supervised by the same professionals, the programmes lasted 6 months and were conducted in similar settings. Besides, this merging strategy has been applied in a previous network study in order to increase the sample size (Bos et al. 2018), and reuse of data from clinical trials is recommended, cost‐effective and useful to test new hypotheses (Ohmann et al. 2017).

Moreover, the minor differences between the two studies (type of training, session duration, age difference, body mass index) were not expected to affect symptom severity, as measured by the PANSS scale.

### 2.4 Evaluation of Psychotic Symptoms

In both Studies 1 and 2, data were collected at baseline and after the 6‐month intervention through interviews conducted by a trained researcher (A.J.R.). Symptoms severity was evaluated using the Positive and Negative Syndrome Scale (PANSS) (Kay et al. 1987) which evaluates positive symptoms (defined as symptoms experienced in addition to reality, such as hallucinations, delusions and disorganised thinking), negative symptoms (referring to an absence or lack of normal mental function like decreased speech fluidity, lack of motivation, and limited abstract thinking), and general psychopathology (providing a parallel measure of the disorder's severity to assess the other symptoms associated with schizophrenia beyond the positive–negative scales). The PANSS includes 30 items: 7 positive, 7 negative and 16 general psychopathology items. This scale has previously been used in network analyses, including studies examining the effects of interventions such as antipsychotics, and has been found to be sensitive to treatment effects (Cai et al. 2024; Esfahlani et al. 2017; Demyttenaere et al. 2022; Santo et al. 2022).

### 2.5 Statistical Analyses

#### 2.5.1 Network Estimation

Associations between symptoms can be represented as a network where symptoms are represented by *nodes* and their relations are *edges*. If two symptoms are associated with each other, they are connected by an edge (line) to indicate a positive or negative association. Line thickness reflects the strength of the association. In the present study, we followed previously published guidelines (Burger et al. 2023).

First, we used the R package *bootnet* with the *estimateNetwork* function to estimate the network structures of the PANSS items before and after the exercise interventions. To decrease the risk of false positive findings and models overfitting, we applied the least absolute shrinkage and selection operator (lasso), and the shrinkage parameter was selected to decrease the extended Bayesian information criterion (EBIC). In our study, given the small sample size, the tuning parameter was set at 0 (Epskamp et al. 2018; Fried et al. 2016).

#### 2.5.2 Comparison Between Network Pre–Post Exercise Intreventions

To investigate whether the network structure was different between pre and post exercise in terms of structure, strength, and edges, we used the *NetworkComparisonTest* package for paired samples with 1000 iterations (van Borkulo et al. 2023). If a difference was detected, a post hoc analysis was performed to locate this difference.

#### 2.5.3 Centrality Estimates

To understand which symptoms were most influential in the network, centrality indices were computed. The centrality indices used were strength, closeness, betweenness and expected influence. Strength (Freeman et al. 1979) represents the weighted sum of all the associations between a given node and all the other nodes. Closeness (Freeman et al. 1979) indicates how close a node is to other nodes in the network. Betweenness (Freeman 1977) indicates how many times a node is the shortest path between two other nodes. Finally, expected influence indicates the node's importance in activating or deactivating other nodes in the network (Robinaugh et al. 2016) and a previous study underlined that in cross‐sectional group‐level networks, this index would be the most appropriate measure of centrality, as it better predicts potential changes in nodes (Spiller et al. 2020). To find a potential difference between pre and post exercise, we statistically compared centrality estimates.

### 2.6 Sensitivity Analysis

Given the small sample size, we performed a sensitivity analysis in which we reduced the number of items to only include the 14 positive and negative symptoms items. The analysis was run with the same parameterization as in the main analysis.

#### 2.6.1 Missing Data

Given missing data cannot be handled in network psychometrics and given the necessity to maintain our sample size, missing data were imputed using multiple imputations by chained equations. This strategy was used given the missingness pattern was found to be completely at random.

## 3 Results

### 3.1 Sample Characteristics

**FIGURE 1:**
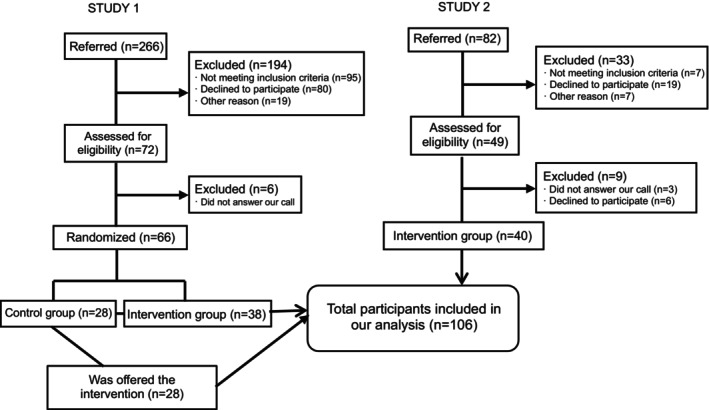
Flowchart of the study.

A sample size of 106 participants (Figure 1) was included and was mainly composed of men (*n* = 60; 56.6%), being Caucasian (*n* = 62; 58.5%), smokers (*n* = 55; 52.9%), with a mean body mass index of 31.2 ± 5.7 kg/m^2^. The main diagnosis was people with schizo‐affective disorders (*n* = 36; 34%) (Table 1).

**TABLE 1:** Sociodemographic characteristics for combined studies.

Combined	Total sample, *n* = 106
Sex, *n* (%)	
Women	46 (34.4)
Men	60 (56.6)
Age (years), *M* (SD)	28.9 ± 6.09
Ethnicity, *n* (%)	
Caucasian	62 (58.5)
Black	23 (21.7)
Others	21 (19.8)
Psychiatric diagnosis, *n* (%)	
Schizophrenia	23 (21.7)
Schizo‐affective disorders	36 (34.0)
Bipolar disorders	30 (28.3)
Major depressive disorder	2 (1.9)
NOS psychosis	15 (14.2)
Smokers, *n* (%)	55 (52.9)
BMI (kg/m^2^)	31.23 ± 5.74
Educational level (completed), *n* (%)	
Primary school	12 (11.3)
Secondary school	43 (40.6)
Diploma or professional studies	9 (8.5)
University diploma	27 (25.5)
Working status, *n* (%)	
Unemployed	66 (62.3)
1–20 h/week	19 (17.9)
More than 20 h/week	21 (19.8)

*Note:* BMI = body mass index, NOS psychosis = not otherwise specified psychosis.

### 3.2 Means and Variation of Positive and Negative Symptoms Scale Items

In the combined sample, based on mean differences, we observed a reduction in severity for 57% of positive (*n* = 4/7), 86% of negative (*n* = 6/7), and 88% of psychopathology symptoms (*n* = 14/16). Symptoms with the largest improvement were P2 (conceptual disorganisation), N6 (lack of spontaneity and flow of conversation), N2 (emotional withdrawal), and G12 (emotional withdrawal). Mean score and standard deviations for all individual PANSS items are in Table 2.

**TABLE 2:** Mean and standard deviation for each PANSS item in combined studies.

PANSS item	Abbreviation	Pre exercise, mean ± SD	Post exercise, mean ± SD
Delusions	P1	1.9 ± 1.2	1.6 ± 0.9
Conceptual disorganisation	P2	2.1 ± 1.2	1.5 ± 0.9
Hallucinatory behaviour	P3	1.9 ± 1.3	1.9 ± 1.4
Excitement	P4	1.5 ± 0.9	1.5 ± 0.8
Grandiosity	P5	1.4 ± 0.8	1.4 ± 0.8
Suspiciousness/persecution	P6	2.0 ± 1.1	1.8 ± 1.0
Hostility	P7	1.3 ± 0.7	1.2 ± 0.5
Blunted affect	N1	2.6 ± 1.4	2.4 ± 1.1
Emotional withdrawal	N2	2.6 ± 1.3	2.2 ± 1.2
Poor rapport	N3	1.8 ± 1.1	1.8 ± 1.1
Passive/apathetic social withdrawal	N4	2.6 ± 1.4	2.3 ± 1.2
Difficulty in abstract thinking	N5	2.5 ± 1.4	2.2 ± 1.4
Lack of spontaneity and flow of conversation	N6	2.3 ± 1.4	1.7 ± 1.0
Stereotyped thinking	N7	1.9 ± 1.1	1.6 ± 0.8
Somatic concern	G1	2.1 ± 1.2	1.8 ± 1.1
Anxiety	G2	2.9 ± 1.2	2.8 ± 1.2
Guilt feelings	G3	2.1 ± 1.3	1.8 ± 1.1
Tension	G4	1.5 ± 0.9	1.4 ± 0.9
Mannerism and posturing	G5	1.9 ± 1.1	1.8 ± 1.0
Depression	G6	2.4 ± 1.4	2.3 ± 1.3
Motor retardation	G7	2.0 ± 1.2	1.7 ± 1.1
Uncooperativeness	G8	1.2 ± 0.6	1.5 ± 0.8
Unusual thought content	G9	1.6 ± 1.0	1.4 ± 0.7
Disorientation	G10	1.4 ± 0.7	1.1 ± 0.4
Poor attention	G11	1.7 ± 1.0	1.6 ± 0.9
Lack of judgement and insight	G12	2.5 ± 1.5	2.1 ± 1.4
Disturbing of volition	G13	2.1 ± 1.1	1.9 ± 1.1
Poor impulse control	G14	1.4 ± 0.8	1.3 ± 0.6
Preoccupation	G15	1.7 ± 0.9	1.8 ± 0.9
Active social avoidance	G16	2.3 ± 1.3	2.0 ± 1.1

*Note:* Level of severity 1 (absent) to 7 (severe); P1–P7 are positive symptoms, N1–N7 are negative symptoms and G1–G16 are psychopathological symptoms.

### 3.3 Network Pre and Post Exercise

**FIGURE 2:**
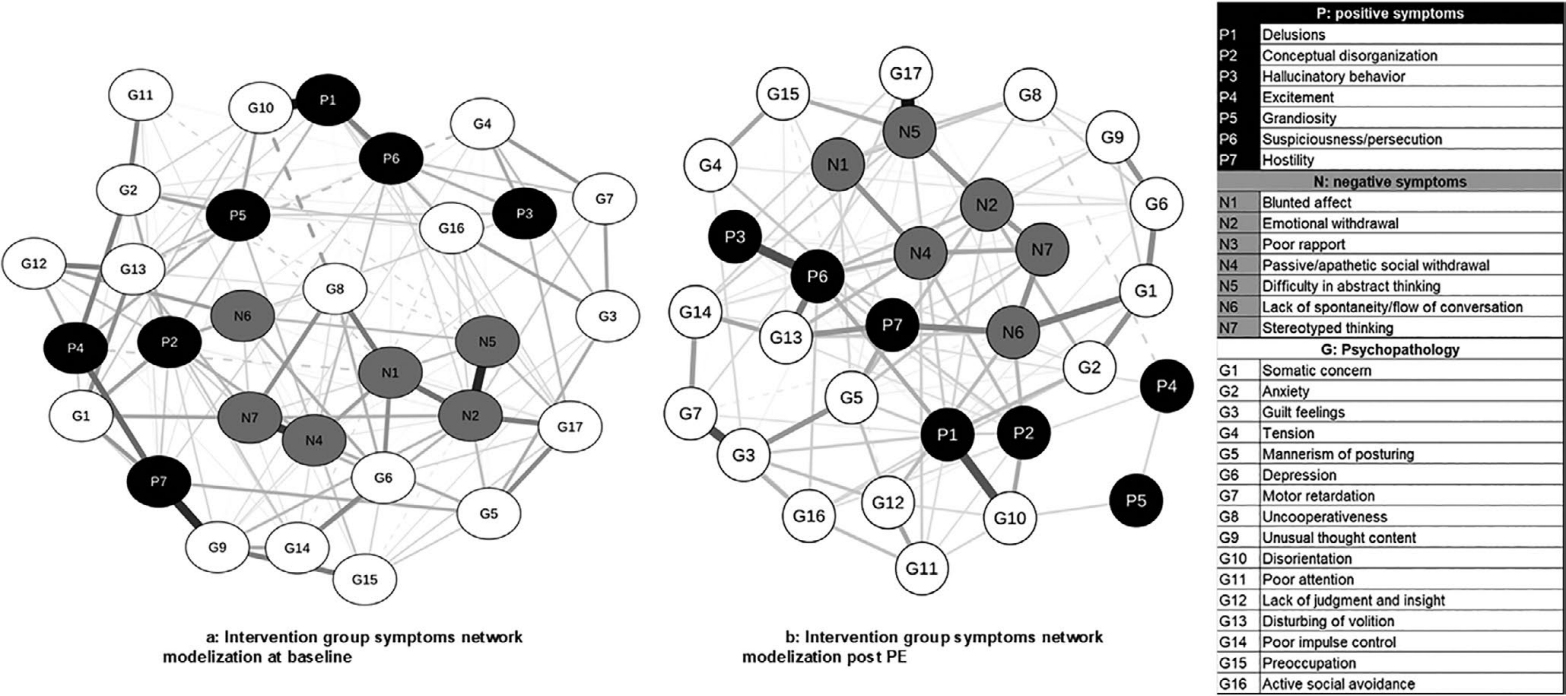
Network modelisation. (a) Intervention group symptoms network modelisation at baseline. (b) Intervention group symptoms network modelization post PE. Positive symptoms are represented by black nodes (dots), negative symptoms by grey nodes and general psychopathology symptoms by white nodes. Solid lines are positive associations, and dotted lines are negative associations. Lines thickness represents the strength of the association, with thicker lines representing stronger association between two connected nodes. G, psychopathology; N, negative symptoms; P, positive symptoms.

Before exercise (Figure 2a), the network was densely connected with several positive connections between the nodes. After exercise (Figure 2b), the network was less densely connected, with fewer connections between symptoms and a decreased strength in the different connections. Those observations suggested that the exercise intervention triggered a disconnection of the network.

When we compared the networks before and after exercise, the network invariance test showed that their structures were statistically different (*M* = 0.38, *p* = 0.005). Given this result, we ran a post hoc to examine which edges were significantly different between the two networks. To decrease the risk of false positives, only edges with an alpha at 0.01 were retained. Results indicated significant differences in 13 different edges before and after exercise (Supporting Information File 1).

Regarding the global strength, reflecting overall network connectivity between the two networks, no difference was found (before exercise: 12.54; after exercise: 8.90, *S* = 3.64, *p* = 0.12).

### 3.4 Network Connectivity

#### 3.4.1 Centrality Indices

**FIGURE 3:**
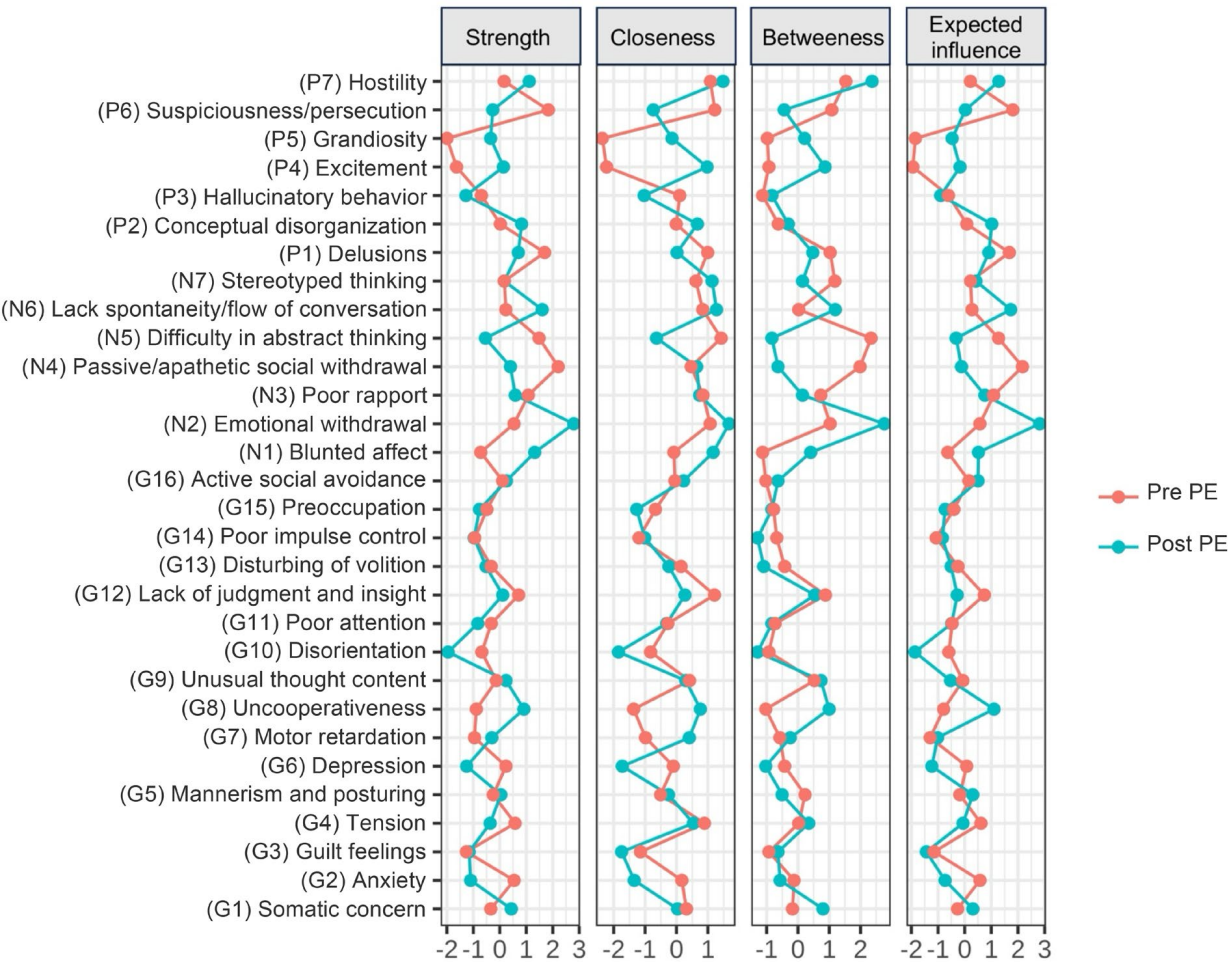
Centrality estimates before and after exercise. Centrality indices before exercise are represented by the blue line and after exercise by the red line.

Before the exercise interventions, symptoms with the highest strength were Emotional withdrawal (N2), Lack of spontaneity and flow of conversation (N6), and Blunted affect (N1) (Figure 3). Symptoms with highest closeness were Emotional withdrawal (N2), Hostility (P7), and Lack of spontaneity and flow of conversation (N6) (Figure 3). Symptoms with the highest betweenness were Emotional withdrawal (N2), Hostility (P7), Lack of spontaneity and flow of conversation (N6) (Figure 3). Symptoms with the highest expected influence were Emotional withdrawal (N2), Lack of spontaneity and flow of conversation (N6), Hostility (P7) (Figure 3).

After the exercise intervention, symptoms with the highest strength were Passive/apathetic social withdrawal (N4), Suspiciousness/persecution (P6), and Delusions (P1) (Figure 3). Symptoms with the highest closeness centrality were Difficulty in abstract thinking (N5), Suspiciousness/persecution (P6), and Lack of judgement and insight (G12) (Figure 3). Symptoms with the highest betweenness were Difficulty in abstract thinking (N5), Passive/apathetic social withdrawal (N4) and Hostility (P7) (Figure 3). Symptoms with the highest expected influence were Passive/apathetic social withdrawal (N4), Suspiciousness/persecution (P6) and Delusions (P1) (Figure 3).

When we compared the centrality indices before and after exercise, significant differences were found in strength and expected influence. Regarding strength, differences were found on the nodes Blunted affect (N1) (*p* = 0.006); Emotional withdrawal (N2) (*p* = 0.003), Lack of spontaneity and flow of conversation (N6) (*p* = 0.01) and Uncooperativeness (G8) (*p* = 0.02). Regarding expected influences, differences were found regarding the nodes Excitement (P4) (*p* = 0.03), Blunted affect (N1) (*p* = 0.04), Emotional withdrawal (N2) (*p* = 0.003), Lack of spontaneity and flow of conversation (N6) (*p* = 0.01), and Uncooperativeness (G8) (*p* = 0.02) (Supporting Information File 2).

### 3.5 Sensitivity Analysis

In this section, we investigated the network using only the 14 positive and negative symptoms items.

#### 3.5.1 Network Connectivity

As in the main analysis, we found that the network was more densely connected before exercise and less connected after exercise (Supporting Information File 3). The network invariance test showed that the structures before and after exercise were not different (M = 0.32, *p* = 0.08). Regarding the global strength, a difference in connectivity was found (before exercise: 6.17; after exercise: 3.76, *S* = 2.41, *p* = 0.027).

#### 3.5.2 Centrality Indices

Before the exercise intervention, symptoms with the highest strength were negative symptoms (i.e., Emotional withdrawal [N2], Lack of spontaneity and flow of conversation [N6]) (Supporting Information File 4). After exercise, symptoms with the highest strength were Emotional withdrawal (N2) and Suspiciousness/persecution (P6).

Before exercise, symptoms with highest closeness were mostly from negative symptoms (Supporting Information File 4) while Delusions (P1) had the highest closeness after exercise. Regarding betweenness, before exercise, prominent symptoms were Emotional withdrawal (N2) and Suspiciousness/persecution (P6) (Supporting Information File 4), while only Delusions (P1) was identified after exercise. Symptoms with the highest expected influence before exercise were Emotional withdrawal (N2), Lack of spontaneity and flow of conversation (N6), and Hostility (P7) (Supporting Information File 4). After exercise, Suspiciousness/persecution (P6) had the highest expected influence.

When we compared the centrality indices before and after exercise, significant differences were found. Regarding betweenness, differences were found on the nodes regarding the nodes Delusions (P1) (*p* = 0.009), and Grandiosity (P5) (*p* = 0.047). Regarding strength, differences were found on Conceptual disorganisation (P2) (*p* = 0.02), Excitement (P4) (*p* = 0.009), Blunted affect (N1) (*p* = 0.01), and Emotional withdrawal (N2) (*p* = 0.004). Regarding expected influence, differences were found on Conceptual disorganisation (P2) (*p* = 0.02), Excitement (P4) (*p* = 0.009), and Emotional withdrawal (N2) (*p* = 0.004).

## 4 Discussion

The main objective of the present study was to explore the mechanisms through which exercise influences psychotic symptoms using a network approach. According to the network theory of mental disorders (Borsboom 2017), a densely connected network indicates increased vulnerability as symptoms are more likely to reciprocally activate and maintain each other. Previous research suggests that network analyses could be used to better understand the relationship, or the effects of exercise in mental health settings (Huong et al. 2025). Our study is thus the first to explore the impact of exercise on psychotic symptoms using a network approach.

Our first hypothesis, the effects of exercise on the PANSS network structure, was supported. Following the intervention, we found that the network was different in terms of structure, being less dense and less connected. This is evidenced by a difference across at least 13 edges in the networks before and after exercise. Notably, among these 13 different edges, several connections involved nodes with the highest strength and expected influence, which could explain the beneficial effects of exercise on symptom severity (Firth et al. 2015; Vancampfort et al. 2012; Rosenbaum et al. 2014). For example, from a clinical point of view, the disappearance of the association between uncooperativeness and conceptual disorganisation symptoms after exercise might be explained by the fact that supervised exercise interventions require cooperation, which in turn probably improves symptom management. Interestingly, previous qualitative research has highlighted that exercise can distract from symptoms and improve thought processes (Hargreaves et al. 2017; Hovland et al. 2023). Those findings are in line with the current literature, and the psychological network theory, which showed significant changes in a mental disorder network structure resulting after a successful treatment course, including antipsychotics (Beard et al. 2016; Bos et al. 2018; Calugi et al. 2021; Hilbert et al. 2020). To another extent, a previous study suggests that the PANSS scale is sensitive in determining treatment effects from a psychological network perspective, notably at the item level (Esfahlani et al. 2017), which confirms the interest of our study.

However, these results should be interpreted in light of the fact that we did not find any difference in terms of global strength between the two networks in our main analysis, while it was the case in the sensitivity analysis. Those results may be explained by the presence of self‐reinforcing loops of symptom activation in the networks of patients with active psychosis, which might play an important role in the maintenance of the disorder. These findings are consistent with the current literature on symptom networks (Calugi et al. 2021; Moura et al. 2021; van Rooijen et al. 2018; Wigman et al. 2015). Another consideration is the sample size and the number of symptoms included in our analysis which influenced our results and explains why we found a difference in the sensitivity analysis. Studies with larger sample sizes showed that participants who responded to pharmacological interventions also found significant differences in the PANSS structure and connectivity (Esfahlani et al. 2017; Li et al. 2025).

Regarding centrality measures more specifically, before the intervention, central symptoms were mostly from positive and negative subscales. According to a recent study in cross‐sectional between‐subject networks (Spiller et al. 2020), expected influence appears as the most appropriate measure of centrality. In the present study, before the intervention, Emotional withdrawal (N2) had the highest value on four indices, including expected influence, indicating its importance in the network connectivity. Lack of spontaneity and flow of conversation (N6) as well as Hostility (P7) also had high values on multiple centrality indices, suggesting that they might play central roles in network activation. After the intervention, central symptoms also came from positive and negative subscales, but the symptoms with the highest value were different, Passive/apathetic social withdrawal (N4) and Suspiciousness/persecution (P6) on expected influence. Difficulty in abstract thinking (N5) and Delusions (P1) nodes were also presenting high centrality values. Interestingly, our results remained quite similar in our sensitivity analysis. Besides, when we statistically compared the centrality values, among nodes being potentially influential in the network, only N1 and N6 were found to be different. These results are particularly interesting as they replicate previous findings showing that both positive and negative symptoms were found to be highly influential, including in studies using repeated cross‐sectional measurement as in our study (Li et al. 2025; Sun et al. 2023). Further, similar nodes were also found to be influential in different studies, including ours (N2, N4, N5, N6, P1), so the specific role of these symptoms should be investigated in future interventions. Otherwise, regarding the other nodes, although significant differences were found, they were not found to be influential in the networks. Overall, from a psychological network point of view, our results showed that exercise has the same mechanisms as other interventions on the PANSS structure, hence explaining its impacts on symptoms as found in several studies (Stubbs et al. 2018). These results are particularly interesting as exercise is an accessible intervention and easy to implement in clinical settings.

Although being an important study to understand the impact of exercise, several limitations should be highlighted. Firstly, even though we used two separate datasets to perform our analysis, our sample size remains small to perform such analyses. Consequently, our study should be replicated with a larger sample size, and with other psychiatric conditions. Secondly, we merged datasets from two studies with specific exercise features, and even though these differences are not known to have an impact on the PANSS structure, we cannot completely exclude that it had an impact on our results. Also, patients included were selected according to specific characteristics such as being physically inactive or being in overweight or obesity, therefore affecting the generalizability of our results to all people with psychosis. Further, centrality outcomes for different symptoms were generated by group‐level data and could not be generalised to individual patients. Constructing a within‐person network would be advised to guide personalised treatment interventions based on the most central symptoms for an individual (Thonon et al. 2020). For instance, ecological momentary assessments of symptoms during exercise intervention could provide large datasets to perform idiographic network analyses (Thonon et al. 2020). Lastly, even though we found an impact of exercise, and our data are based on intervention trials, our data were cross‐sectional, so it is not possible to draw causation. So, future studies should use repeated assessments to better understand the impact of exercise on symptoms using the network approach.

In conclusion, this physical activity study is the first investigating symptom networks in a population of people with psychosis. Using standardised procedures, we demonstrated how exercise intervention impacts the symptom network density and structure. The present study sheds light on an interesting first mechanism of action of physical activity on the psychotic symptom network that still requires more exploration.

## Funding

This work was supported by Fondation de l'Institut universitaire en santé mentale de Montréal and FRQS research fellowship and doctoral fellowship.

## Ethics Statement

The protocols have been approved by the ethics committee of the CHUM research center ##10.255 and 16.399.

## Conflicts of Interest

The authors declare no conflicts of interest.

## Supporting information


**Data S1:** eip70124‐sup‐0001‐supinfo.docx.

## Data Availability

The data that support the findings of this study are available from the corresponding author upon reasonable request.
