# Supplementary material for: The Impact of Exercise Interventions on the Network Structure of Psychotic Symptoms: Analysis From Two Clinical Trials
Source: Early Interv Psychiatry. 2026 Jan 7;20(1):e70124. doi: 10.1111/eip.70124 (PMC12780304; doi:10.1111/eip.70124)
Supplement: Supplementary file 1 — Data S1: eip70124‐sup‐0001‐supinfo.docx. [file EIP-20-0-s001.docx]

**Supplementary files**

1. Edge differences in the networks before and after exercise intervention
2. Statistical differences between centrality indexes before and after exercise intervention
3. Network of psychotic symptoms
4. Centrality indices before and after exercise intervention

Supplementary file 1. **Edge differences in the networks before and after exercise**

Notes: P = positive symptoms, N = negative symptoms, + = connection between the two nodes present before exercise; ++ = connection between the two nodes present after exercise; ≡ = connection between the nodes before and after exercise.

Supplementary file 2. **Statistical differences between centrality indexes before and after exercise**

<

Supplementary file 1. Statistical differences between centrality indexes before and after exercise intervention

| SYMPTOMS | **CLOSENESS** | **BETWEENNESS** | **STRENGTH** | **EXPECTED INFLUENCE** |
| --- | --- | --- | --- | --- |
| **P1** | NA | 0.57 | 0.98 | 0.98 |
| **P2** | 0.12 | 0.89 | 0.13 | 0.13 |
| **P3** | NA | 0.47 | 0.75 | 0.75 |
| **P4** | NA | 0.12 | 0.06 | 0.03 |
| **P5** | NA | 0.14 | 0.06 | 0.09 |
| **P6** | 0.99 | 0.23 | 0.16 | 0.16 |
| **P7** | 0.32 | 0.95 | 0.29 | 0.29 |
| **N1** | 0.09 | 0.32 | 0.01 | 0.04 |
| **N2** | 0.11 | 0.59 | 0.00 | 0.00 |
| **N3** | 0.31 | 0.63 | 0.61 | 0.64 |
| **N4** | 0.17 | 0.08 | 0.41 | 0.07 |
| **N5** | 0.97 | 0.02 | 0.42 | 0.47 |
| **N6** | 0.11 | 0.74 | 0.01 | 0.01 |
| **N7** | 0.16 | 0.31 | 0.39 | 0.39 |
| **G1** | NA | 0.63 | 0.17 | 0.26 |
| **G2** | 0.8 | 0.73 | 0.55 | 0.53 |
| **G3** | NA | 0.28 | 0.43 | 0.78 |
| **G4** | 0.41 | 0.96 | 0.97 | 0.97 |
| **G5** | 0.20 | 0.39 | 0.27 | 0.26 |
| **G6** | 0.85 | 0.48 | 0.71 | 0.49 |
| **G7** | 0.05 | 0.63 | 0.09 | 0.24 |
| **G8** | NA | 0.08 | 0.02 | 0.02 |
| **G9** | NA | 0.95 | 0.17 | 0.78 |
| **G10** | NA | 0.33 | 0.78 | 0.48 |
| **G11** | NA | 0.92 | 0.70 | 0.53 |
| **G12** | NA | 0.58 | 0.70 | 0.79 |
| **G13** | 0.36 | 0.53 | 0.48 | 0.70 |
| **G14** | NA | 0.40 | 0.38 | 0.38 |
| **G15** | NA | 1.00 | 0.54 | 0.76 |
| **G16** | 0.12 | 0.71 | 0.31 | 0.30 |

**Notes:** data are p values; P1 to P7 are positive symptoms, N1 to N7 are negative symptoms, G1 to G16 are psychopathological symptoms.

Supplementary file 3: **Network of psychotic symptoms**


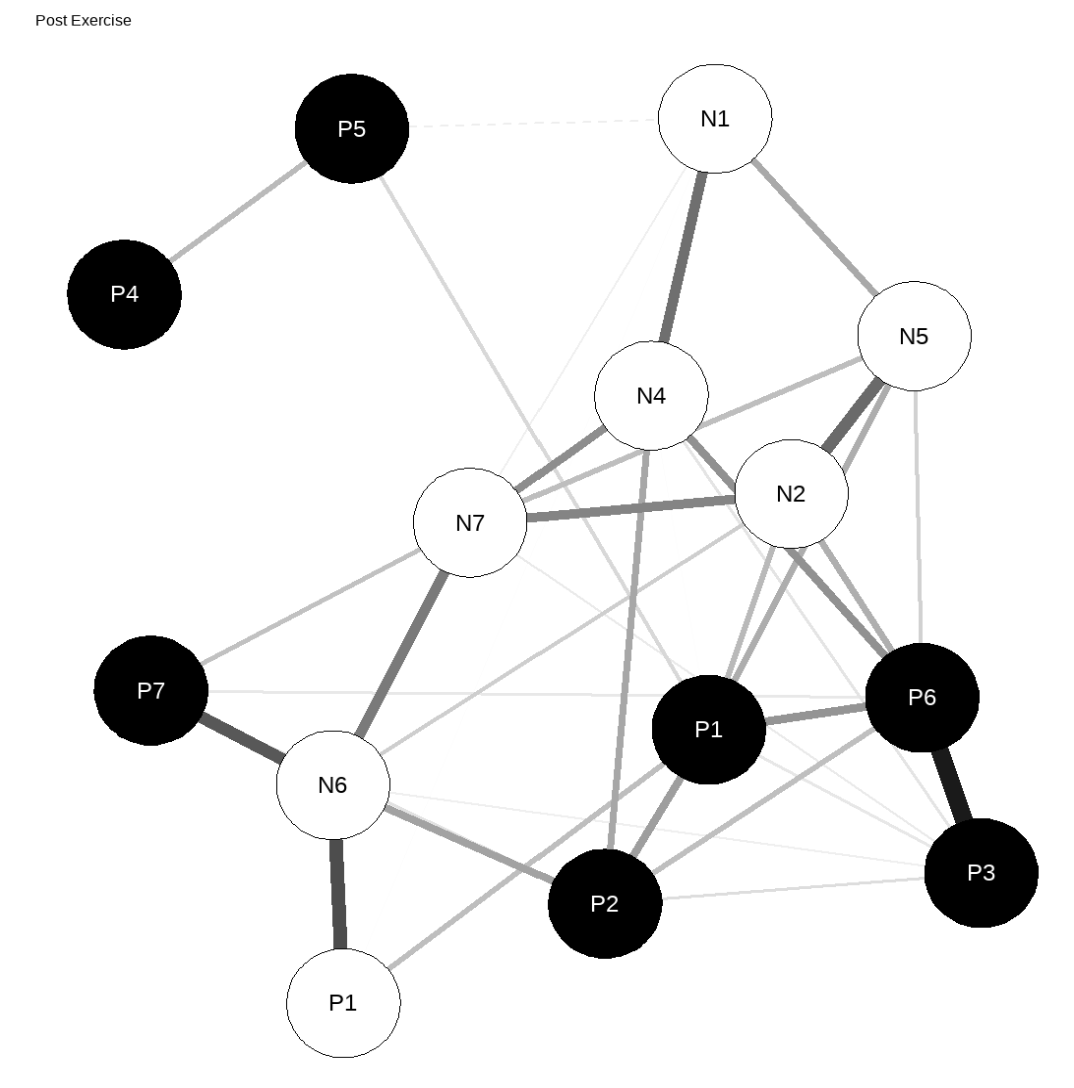

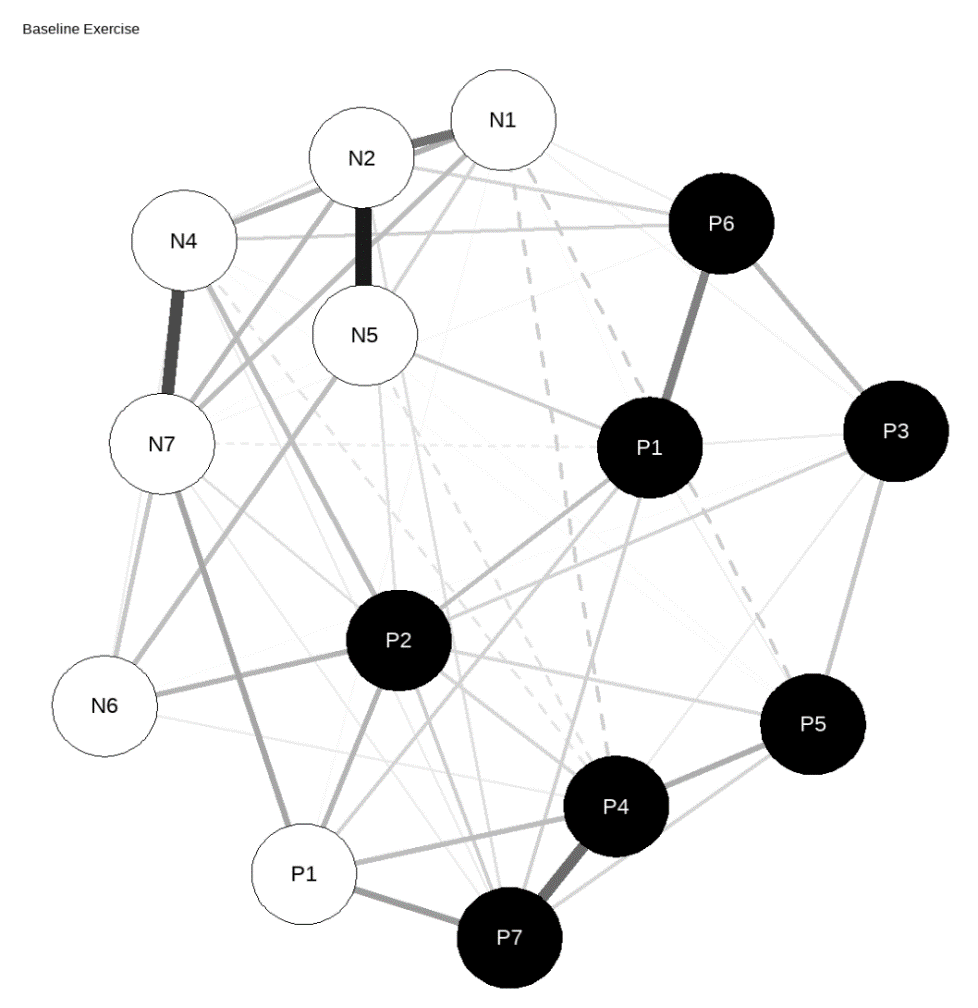
**Notes:** Positive symptoms are represented by black nodes (dots), and negative symptoms by white nodes. Solid lines are positive association, and dotted lines are negative associations. Lines thickness represent the strength of the association, with thicker lines representing stronger association between two connected nodes.


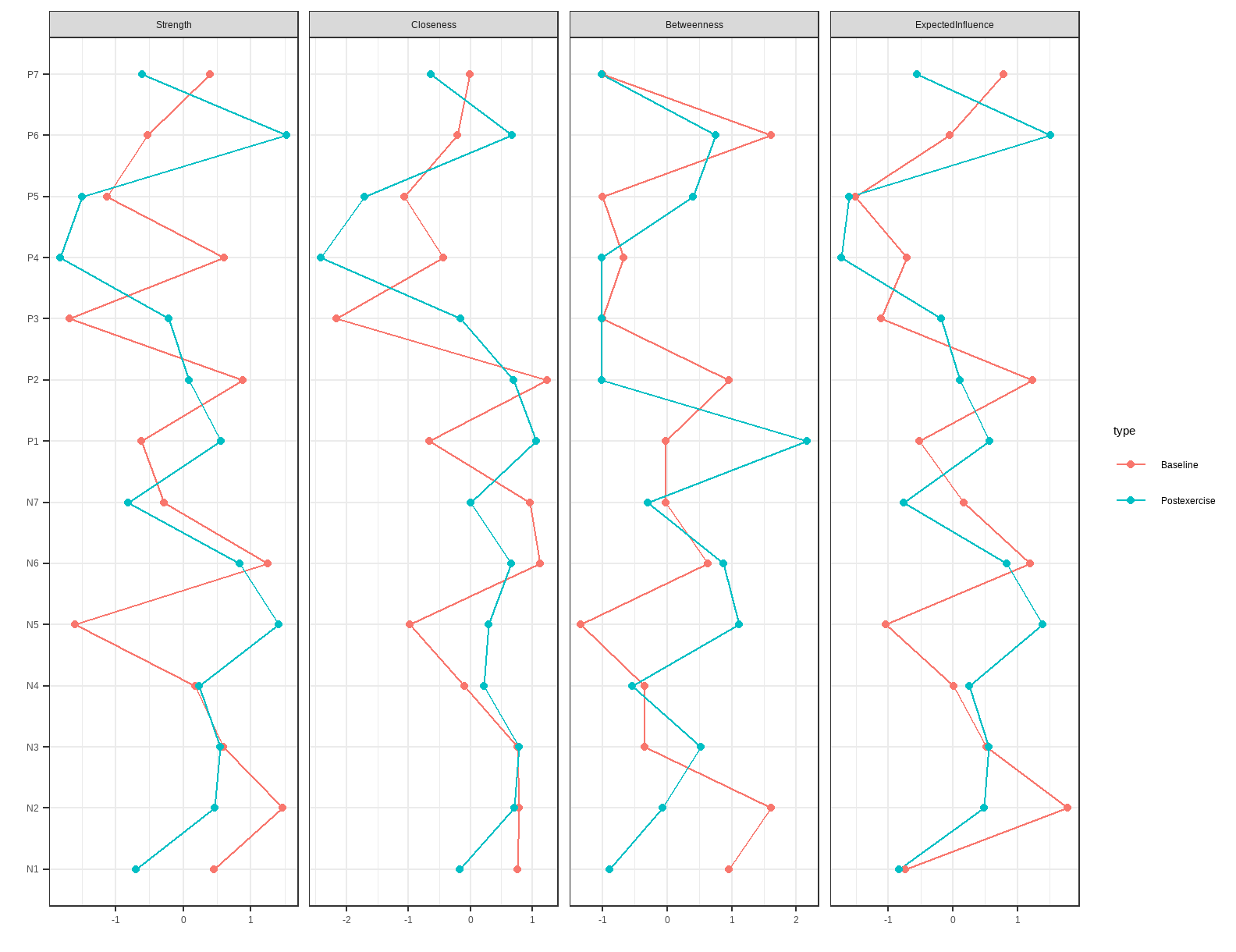
Supplementary file 4: **Centrality indices before and after exercise intervention**

**Notes:** The red line represents centrality indexes before exercise, and the blue line after exercise.
